# Predictive value for cerebrospinal fluid Alzheimer's disease profile of different measures of verbal episodic memory in patients with MCI

**DOI:** 10.1038/s41598-024-62604-z

**Published:** 2024-05-28

**Authors:** Nicola Salvadori, Edoardo Guido Torrigiani, Federico Paolini Paoletti, Elena Chipi, Chiara Montanucci, Claudio Verderosa, Elisa Siena, Daniela Fruttini, Lucilla Parnetti

**Affiliations:** 1 Centre for Memory Disturbances, Lab of Clinical Neurochemistry, Section of Neurology, Department of Medicine and Surgery, University of Perugia, Perugia, Italy; 2 Section of Endocrinology and Metabolism, Department of Medicine and Surgery, University of Perugia, Perugia, Italy

**Keywords:** Verbal episodic memory, Mild Cognitive Impairment, Alzheimer’s disease, CSF biomarkers, A/T/(N) classification, Neuroscience, Psychology, Biomarkers, Neurology

## Abstract

Neuropsychological evidence of memory impairment represents the main feature of the clinical onset of typical Alzheimer’s disease (AD). Rey’s Auditory Verbal Learning Test (RAVLT) and Logical Memory (LM) are two tests both assessing verbal episodic memory, widely used in clinical practice. Our aim was to investigate the added value of their combined use in predicting cerebrospinal fluid (CSF) AD biomarkers positivity in a retrospective consecutive series of patients with mild cognitive impairment (MCI). 169 MCI patients were included. For all of them neuropsychological assessment and CSF analysis were available. According to CSF A/T/(N) profile, 109 were defined as MCI due to AD (A+T+), and 60 were non-AD MCI (A−T−). Logistic regression model and receiver-operating characteristic (ROC) curves were analyzed to evaluate the discriminatory power of single and combined sub-measures between AD and non-AD patients. The combination of RAVLT-del with LM could acceptably discriminate the two groups (AUC: 0.69, CI 95% 0.617–0.761, sens: 0.75, spec. 0.58, p < 0.001), while the single tests did not show sufficient discriminative performance. Our study shows that the combination of RAVLT delayed recall with LM better predicts the biological AD diagnosis (A+T+), showing a good discriminative power between MCI-AD from non-AD MCI. Since RAVLT and LM assess different components of verbal episodic memory, they should be considered as complementary, rather than interchangeable, tests.

## Introduction

Mild Cognitive Impairment (MCI) is a clinical syndrome characterized by cognitive decline with respect to a previous normal level and with respect to normative mean values, defined as a performance of 1.5 SD below age- and education- matched controls in one or more cognitive domains^1,2^. From a neuropsychological point of view, the original criteria were based on a preeminent memory impairment, self-reported by the patient and objectively detected with cognitive measures^1^.

Although MCI has been originally conceptualized as a transitional stage between normal cognition and dementia^1,3,4^, it represents a heterogeneous condition with different possible underlying etiologies, i.e., neurodegenerative, vascular, psychiatric, multi-factorial^5,6^. The MCI picture may represent the consequence of several concomitant factors, and different trajectories can be observed over time^7^. Patients with MCI can remain stable, also reverting into normal cognition, which raises some issues about the correct classification of MCI. Reversion into normal cognition could be the consequence of misdiagnosis, also depending on the neuropsychological criteria adopted. To avoid such misclassification, current neuropsychological criteria for MCI recommend the use of at least two tests for each cognitive domain. Several studies found that the use of original criteria based on impairment in one test (*one-test approach*) may lead to a high proportion of false positives^8,9^ and, to a lesser extent, false negatives^10^. As MCI is a clinical syndrome and not a diagnostic entity per se, the investigation of in vivo biomarkers is crucial to reach an etiological diagnosis, i.e., at first to rule out the presence of Alzheimer’s Disease (AD). By now, definition of AD has been shifted from a syndromal to a biological construct. As amyloidosis and tauopathy are the neuropathological hallmarks of AD, the positivity of in vivo biomarkers reflecting amyloidosis and tauopathy, i.e., reduced amyloid-β42/β40 ratio (Aβ42/Aβ40) and increased levels of phosphorylated tau (p-Tau) in cerebrospinal fluid (CSF), allows to define AD independently from the clinical stage. According to the categorical classification of biomarkers proposed by the National Institute on Aging and Alzheimer’s Association (NIA-AA) research framework, namely the A/T/(N) system, “A” refers to amyloidosis, “T” refers to tauopathy and “N” refers to neurodegeneration. AD is defined by biomarkers evidence of cerebral amyloidosis (A+) and tauopathy (T+) (A+/T+ profile)^11–13^.

Patients affected by amnestic syndrome of hippocampal type^14–18^ typically show a characteristic pattern of memory impairment: a flattened learning slope, a poor free recall and a scarce benefit from the facilitation by cues and false recognitions^15^. This profile is also defined as *temporo-limbic amnesia*. Memory deficits observed in the early phases of AD are related to the damage of the neuroanatomical underpinnings of episodic memory^19,20^.

Declarative memory, encompassing episodic, semantic and autobiographical aspects of long-term memory, strongly depends on the intertwining functional activation of different brain regions within the medial temporal lobe^21,22^ Episodic memory is highly focused on spatial and temporal details of events, being defined as *context-rich memory*^23^. Conversely, *context-free* memories are not dependent from the context of learning^24^. Past models of episodic memories (*standard systems consolidation theory,* SSCT) assigned a preeminent role to the hippocampus for consolidation of new traces, i.e., new memories being temporally-dependent by hippocampal activity and gradually migrating to neocortex for long-term storage^25^. Current neurocognitive models assign a crucial role to hippocampus in each phase of learning and retrieval (i.e., *multiple trace theory*); at the same time, dissociable roles are shown for *familiarity* and *recollection*: the former allows recognition of context-free traces (i.e., objects, faces, places) and semantic aspects, and is based on neocortical and para-hippocampal regions; the latter refers to the ability to retrieve complex and detailed memories and is highly dependent from hippocampus and posterior network^23,25^.

A hierarchical model of declarative memory may account for the temporally ordered gradient of memory decline in AD. According to this model, a dissociation can be observed between a ventral system, devoted to object recognition and scenes discrimination that mediates *context-free* memories, and a dorsal network, sub-serving spatial and relational aspects, involving neocortical regions and crucial for *context-rich* memory^23^. Specifically, the former system includes the anterior hippocampus, perirhinal and entorhinal cortex, lateral and anterior temporal neocortex (temporal pole), highly involved in semantic processes and memory for single objects, and firstly affected by neurofibrillary tangles (NFTs) deposition in AD continuum^26^. Conversely, the latter posterior network includes the body of the hippocampus, posterior parahippocampal cortex, posterior midline regions (i.e., posterior cingulate and precuneus) and medial prefrontal cortex. This network may allow the association of relationships among different items and is crucial for contextual memories, and seems to be affected later by NFTs deposition^21,23^. Several brain regions beyond the MTL are crucial for the efficient performance at memory tasks, that requires the intervention of other non-memory processes^27^. Since the interplay between prefrontal and medial temporal regions is crucial to bind single units of information within an associative link during learning and retrieval^28^, both the AD pivotal neuropathological features—amyloidosis and tauopathy—play a role in the occurrence of episodic memory impairment.

Phases and components of verbal episodic memory are guaranteed by distinct brain regions^22,27^. Immediate recall relies on the activation of inferior parietal lobule and temporal pole, as mainly mediating verbal-auditory working memory and semantic processing, respectively; delayed recall is uniquely associated with hippocampal integrity and the recognition phase of RAVLT is highly dependent from integrity of extra-hippocampal regions (perirhinal and entorhinal cortex, subcortical and prefrontal regions)^25,29,30^. The posterior cingulate cortex (PCC), precuneus, and posterior inferior parietal lobule have been linked to successful memory encoding and retrieval in word lists^22^ although prefrontal cortex also plays a role.

In order to accurately capture the features of the *temporo-limbic* amnesia, it is crucial, in clinical setting, to use reliable neuropsychological measures of episodic memory. Word list recall and story recall are commonly used paradigms for verbal episodic memory assessment. The most traditional word list paradigm is the Rey Auditory Verbal Learning Test (RAVLT)^31,32^, and the prose recall of a story (*story recall*) is usually assessed by the Babcock short story^33^ or the short story of Anna Pesenti^34^; the latter memory measures are paradigms of *Logical Memory Test*. Previous studies reported that these tasks are not interchangeable^35,36^, and the use of only one of the two paradigms increases the risk of under- or over-estimation of amnestic MCI^7^.

Significant differences between the two measures are observed according to the degree of involvement of cognitive processes, namely executive functions^18,37^. For example, the recall of a short story requires the verbal information to be put into a structured, organized context, leading to less effort in the encoding and retrieval of the verbal content. Conversely, a higher involvement of executive functions is required to learn and recall unrelated words in a long list, especially by means of the implementation of efficient strategies for encoding. In particular, subjects with prominent executive impairment, i.e. those affected by fronto-temporal dementia (FTD), typically have worse performances in this memory paradigm than short story recall^18,37,38^. Conversely, patients with AD can show significant difficulties with both word list and recall of a short structured story, reflecting the characteristic hippocampal amnestic syndrome with scarce benefit of facilitation. The lack of improvement by contextual cues strongly orients toward a “pure” amnestic syndrome with underlying damage of medial temporal lobe. Accordingly, a combination of the two memory tests can be more informative with AD patients^38^. Some studies support the combined use of the RAVLT and short story recall to improve the accuracy in predicting clinical conversion from MCI to dementia over time^39,40^. Stable MCI patients are found to show poor free recall of a word list despite a performance similar to healthy controls on the recall of a short story, while those who converted to dementia displayed a significant forgetting in both tests, showing no benefit of semantically organized structure of the story^18,38^.

In sum, some evidences indicate that the combination of list learning with story recall can be of help in detecting episodic memory impairment. However, the predictive value of these neuropsychological measures can be assessed if available the pathophysiological causes of MCI, with special interest on MCI due to AD. The aim of the present study is to investigate the role of the sub-measures of these two memory tests in predicting the CSF AD biomarkers positivity in a retrospective, consecutive cohort of well-characterized MCI patients, referring to our Memory Clinic for diagnostic assessment.

## Patients and methods

### Study population

Our population consisted of 169 patients referring to the Centre for Memory Disturbances, Section of Neurology, Perugia University Hospital from 2016 to 2022. Each patient underwent clinical evaluation, comprehensive neuropsychological assessment and lumbar puncture for CSF profiling according to the A/T/(N) system^13^. All patients with Mini-Mental State Examination adjusted score above the cut-off (≥ 23.8)^41^, impairment of at least one cognitive domain defined as Equivalent Score (ES) = 0, and a Clinical Dementia Rating scale global score = 0.5 were classified as MCI.

### Neuropsychological testing

Each patient underwent a baseline comprehensive neuropsychological assessment, including: Mini-Mental State Examination (MMSE)^41^ for assessing global cognition; Trail Making Test (TMT) part A and B^42^ and the Frontal Assessment Battery (FAB)^43^ for assessing attention and executive functions; the Rey’s Auditory Verbal Learning Test (RAVLT, with sub-measures of immediate and delayed recall, true and false recognitions)^32^ and the Short story test of “Anna Pesenti” (immediate and delayed recall)^34^ for the assessment of verbal episodic memory; the digit span forward and backward^44^ for the assessment of short-term verbal memory and working memory; 1-min phonemic fluency^32^ and category word fluency^34^ for assessing language; copy of drawings with and without landmarks from the Mental Deterioration Battery (MDB)^32^ and the Clock Drawing Test (CDT)^45^ for the assessment of visuo-constructional abilities; the Raven Progressive Matrices (MP’47)^46^ for the assessment of logical-perceptual reasoning. The neuropsychological assessment also included the Clinical Dementia Rating Scale (CDR)^47^ for staging functional decline. MCI subtype was classified according to neuropsychological profile observed as follows: amnestic-single-domain MCI (a-sd MCI) if only the memory domain was impaired; amnestic-multi-domain MCI (a-md MCI) if memory and at least another domain were involved; if memory domain was spared but other domains were impaired, patients were classified as non-amnestic MCI (na-MCI), including both the non-amnestic single domain MCI (na-sd-MCI) and non-amnestic multi-domain MCI (na-md-MCI) subtype^6^.

### Sub-measures of verbal episodic memory tests

Among the neuropsychological tests, we analyzed the scores obtained at RAVLT and LM. The immediate recall of RAVLT (RAVLT-imm) consists in the total number of words recalled during the 5 learning trials (0–75). The delayed recall of RAVLT (RAVLT-del) consists in the number of words spontaneously recalled after the 15-min interval (0–15). The True and False Recognitions (RAVLT-true, RAVLT-false) are scored as the number of true hits (0–15) and false alarms (0–31) during the recognition trial. The short story recall (LM) total score was calculated as the average between the immediate and the 10-min delayed recall (0–28).

### Lumbar puncture and CSF analysis

Lumbar puncture (LP) was performed according to international guidelines^48^. Briefly, 10–12 mL of CSF were collected in sterile polypropylene tubes and centrifuged at room temperature for 10 min (2000 × g). Aliquots (0.5 mL) were frozen at − 80 °C. CSF analysis included routine chemical-physical parameters (glucose and total proteins) and cell count. Blood-contaminated samples, i.e., more than 50 red cells/μL were excluded from the analysis. Aβ40, Aβ42, t-Tau and p-Tau were analyzed using Lumipulse G600-II fully automated chemiluminescent enzyme immunoassay system in our Lab. of Clinical Neurochemistry. The CSF A/T/(N) profile was subsequently considered for all patients, and A+/T+ profile was considered as CSF AD-like profile. According to the cut-off values calculated in our Lab^49^, “A+” corresponds to a CSF Aβ42/40 ratio < 0.072, and “T+” corresponds to a CSF phospho-tau > 50 pg/mL.

### Ethical declaration

Since 2008, CSF collection for the early diagnosis of Alzheimer’s disease is routinely performed in our Center, as approved by the local Ethics Committee (CER Umbria, Protocol N° 19369/08/AV, registry N. 1287/08, date: 9 October 2008). All patients gave their written informed consent for the study participation. The research was performed in accordance with the Declaration of Helsinki.

### Statistical analyses

Statistical analyses were conducted via Statistical Package for the Social Sciences (IBM SPSS). We first analyzed the differences between the two cohorts in age, MMSE, RAVLT and Short story sub-measures.

Wilcoxon Mann–Whitney U test was used to determine the power of each RAVLT and Short story sub-measure to discriminate between patients in the AD group (MCI-AD) from the subgroup with negative biomarkers profile (non-AD MCI).

ROC curves were generated to estimate the power of each RAVLT and Short story sub-measure to discriminate between patients with MCI-AD from non-AD MCI.

A stepwise logistic regression model was used to investigate the power of combined RAVLT and Short story sub-measures to discriminate between patients with MCI-AD from non-AD MCI.

We also investigated the predictive value of the total impairment in multiple sub-measures, calculated as cumulative score derived by the number of memory sub-measures impaired (0–5), in which a score of 1 is attributed to performances below current cutoffs and 0 to measures within normal range, and the biological diagnosis of AD based on CSF biomarkers. This index has been proposed as exploratory analysis that holds together all the sub-measures of the two episodic memory tests (RAVLT-imm, RAVLT-del, RAVLT-True, RAVLT-False, LM). A p-value < 0.05 was considered statistically significant. All analyses were performed using IBM SPSS V.25.0.0.

## Results

### Demographical, clinical and CSF features

Demographic data, clinical features, and biomarkers values are listed in Table 1.

**Table 1:** Demographical features and neuropsychological measures in MCI-AD and non-AD MCI groups. Data are expressed as mean ± standard deviation.

	MCI-AD	non-AD MCI	p-value
Demographical features			
N	109	60	–
sd-aMCI (N.)	19	10	–
md-aMCI (N.)	82	32	–
Age (y)	72.7 ± 5.2	71.7 ± 5.9	0.204 (n.s.)
Education (y)	10.78 ± 4.35	10.79 ± 4.32	0.392 (n.s.)
CSF biomarkers			
Aβ42/Aβ40	0.47 ± 0.12	0.97 ± 0.29	**0.001**
p-Tau	112.66 ± 57.52	43.02 ± 12.89	**0.001**
t-Tau	757.22 ± 350.70	304.57 ± 112.21	**0.001**
Neuropsychological measures			
MMSE	24.62 ± 2.74	26.3 ± 1.91	**0.001**
RAVLT-imm	23.8 ± 6.83	27.07 ± 7.14	**0.001**
RAVLT-del	2.15 ± 1.98	3.43 ± 2.22	**0.0001**
RAVLT-true	11.34 ± 3.21	12.05 ± 2.28	0.417
RAVLT-false	6.96 ± 5.9	4.62 ± 5.23	**0.01**
LM	5.6 ± 4.02	7.78 ± 4.25	**0.001**

LM: logical memory- short story recall; md-aMCI: multi domain-amnestic MCI, RAVLT-imm: Rey’s auditory verbal learning test—immediate recall; RAVLT-del: Rey’s auditory verbal learning test—delayed recall; RAVLT-true: Rey’s auditory verbal learning test—true recognitions; RAVLT-false: Rey’s auditory verbal learning test—false recognitions; sd-aMCI: single domain-amnestic MCI.

Significant values are in bold.

#### Whole MCI group

The mean age of the whole MCI group (n. 169; 73 M, 96 F) was 72.34 ± 5.35 years, with mean years of education of 10.56 ± 4.3. Concerning the clinical subtype of MCI patients, the majority of them showed an amnestic phenotype (n. 140, 83%). Among aMCI, the wide majority (113/140, 80%) showed multiple domain, being classified as md-a-MCI. This subgroup represented the most prevalent in the whole cohort (67%).

#### MCI-AD vs. non-AD MCI

Based on CSF profile, 109 were classified as MCI due to AD (A+T+ , 64.5%), and 60 as non-AD MCI (A−T−, 35.5%). No differences were found between the two groups in terms of age, education and gender. With respect to the distribution of the clinical subtypes (amnestic/non amnestic, single/multiple domain), we found that the great majority of MCI-AD (91%), and more than half of the non-AD MCI group (67%) showed memory impairment.

By applying the Chi-Squared test, we found a strong association of memory impairment with CSF AD profile (Χ^2^ = 15.400, p = 0.0001). Both single- and multi-domain amnestic MCI patients showed a moderate predictive value for CSF AD profile (AUC = 0.693, SE = 0.0411, 95% CI 0.617–0.761, sens (%) = 75.5, spec (%) = 58.3).

By using the Wilcoxon Mann–Whitney U test, we found that MCI-AD patients showed worse scores on MMSE, RAVLT-imm, RAVLT-del, RAVLT-false and LM with respect to non-AD MCI (p < 0.05, see Table 1).

### Predictive value of neuropsychological measures

**Figure 1:**
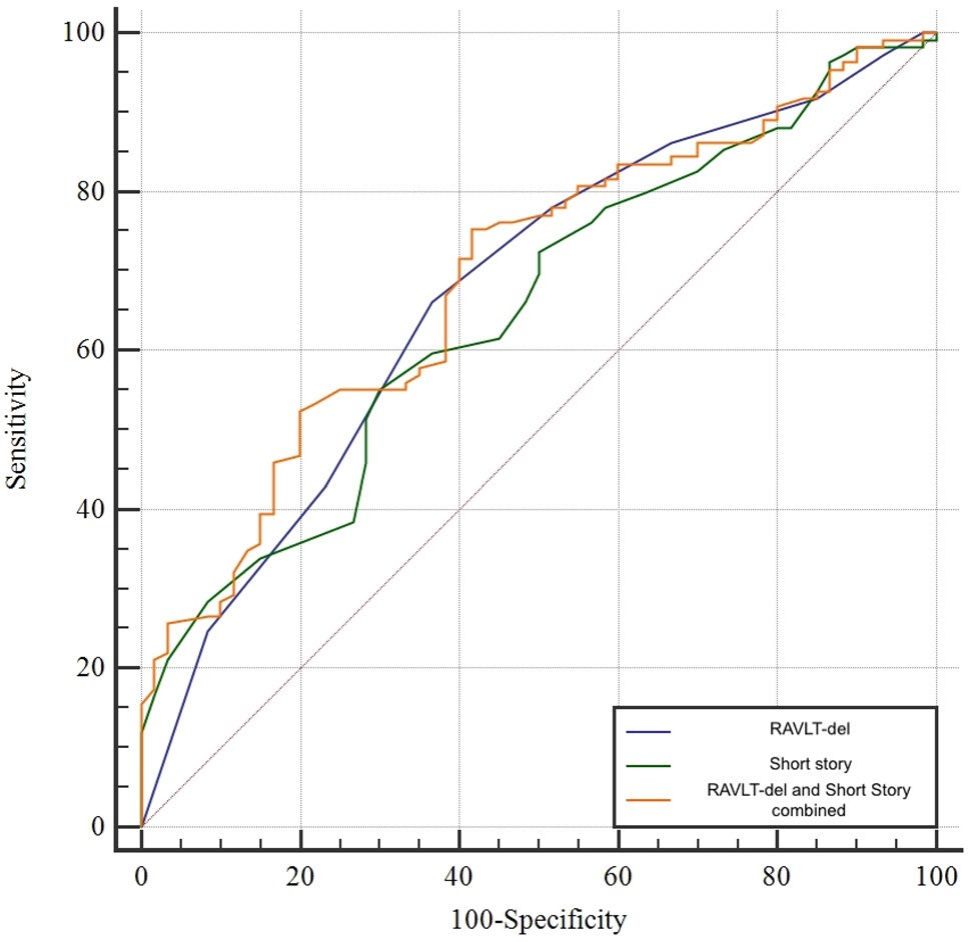
Comparison between ROC curves generated to discriminate between patients with an A+/T+ CSF profile and non-AD patients: RAVLT-del alone, Short story recall (LM) alone and the one generated with the combined use of both sub-measures.

We used a stepwise logistic regression model to investigate the predictive value of the memory tests sub-measures for AD diagnosis. RAVLT-del and LM reached statistical significance in predicting the AD diagnosis (A+/T+) (RAVLT-del: p = 0.0091; LM: p = 0.05). The combination of both measures showed the best performance in discriminating the two groups (AUC: 0.69, CI 95% 0.617–0.761, p < 0.001) (see Fig. 1).

We also applied stepwise logistic regression model to evaluate the association of each memory test with any single biomarker. We thus found that positivity of amyloidosis (A+) is predicted by RAVLT-false (p = 0.04) and LM (p = 0.005). The combination of LM and RAVLT-false gives the best predictive value (p < 0.001). RAVLT-del predicts positivity of tauopathy (T+, p = 0.004) and neurodegeneration (N+, p = 0.003).

We also explored the predictive value of a cumulative score obtained as the number of impaired memory sub-measures (RAVLT-imm, RAVLT-del, RAVLT-true, RAVLT-false, LM), calculated as 0 (normal with respect to cognitively unimpaired subjects) or 1 (impaired) according to the age- and education-adjusted raw scores, on CSF AD profile. According to the ROC analysis, the cumulative score predicted the positivity of each biomarker. Higher cumulative scores discriminated A+ vs A− patients (AUC = 0.730, Χ^2^ = 22.997, p < 0.0001, 95% CI 0.657 to 0.796); T+ vs T- patients (AUC = 0.682, Χ^2^ = 16.797, p = 0.0001, 95% CI 0.606–0.752); N+ vs N− patients (AUC = 0.651, Χ^2^ = 11.400, p = 0.0007, 95% CI 0.574–0.723). The stepwise logistic regression model showed RAVLT-del and LM sub-measures as significant variables.

## Discussion

The aim of the present study was to investigate the predictive value of two traditional tests assessing verbal episodic memory (the RAVLT and the short story recall, LM) and their distinct sub-measures on CSF AD profile in a retrospective, consecutive cohort of MCI patients including MCI AD and non-AD MCI. As expected, the two subgroups significantly differed in the mean scores on MMSE, RAVLT-imm, RAVLT-del, RAVLT-false and LM.

In our cohort, 93% of patients with MCI due to AD showed deficits in memory domain. Of them, 18% showed single domain amnestic MCI, vs. 82% showing multi-domain amnestic MCI. These results are in line with previous evidence indicating that multi-domain amnestic profile is much more common than single-domain amnestic phenotype^50–52^.

Our main finding is that the combination of two distinct verbal episodic memory tests reached the highest sensitivity in predicting CSF AD profile, i.e. A+/T+. Specifically, the RAVLT-del and LM better predicted CSF AD profile, with a fair discriminative power between MCI-AD and non-AD MCI. Such results are in line with previous evidence of a specific neuropsychological pattern of episodic memory impairment in patients with MCI due to AD. Subjects with a “pure” amnestic syndrome of hippocampal type showed a consistent rate of forgetting in a word list and in a story recall, as reflected by impaired delayed recall at both tests^18,53^. However, poor delayed recall per se is not specific for AD and the use of a single cognitive measure may lead to misclassification of MCI. In fact, a single-measure/single-test approach, as well as the use of a single test to assess a cognitive domain, has been largely criticized as it may lead either to under- or over-estimation of cognitive decline^8^.

Free and Cued Selective Reminding Test (FCSRT) is another widely used tool for the assessment of hippocampal memory, based on encoding specificity paradigm^14,24^. Total (free + cued) recall of FSCRT has been found to be associated with anterior MTL atrophy^24^ and CSF AD profile^54^. Due to the intrinsic paradigm used, the FCSRT is not properly an *episodic memory* test^24^. These findings support the importance, in clinical practice, of the combined use of memory tests giving complementary information, such as LM and RAVLT.

When considering the association of neuropsychological measures with the single CSF biomarkers, we observed different behaviors. The delayed recall of a word list correlated with CSF p-Tau, while the short story recall was found to be associated with CSF Aβ42/40. Previous studies investigating the association of specific measures of episodic memory with CSF AD biomarkers reported contradictory results^55–61^.

The tendency to produce false positives during recognition tasks is a key feature of the amnestic syndrome of hippocampal type underlying the episodic memory deficits in AD. Amnestic MCI patients showing impaired free delayed recall associated with a recognition deficit may have a more profound consolidation deficit (“encoding/storage” pattern), distinct from those with impaired delayed recall with spared recognition (“retrieval” deficit)^50^. Such syndrome characterizing the clinical onset of typical AD encompasses the lack of benefit by cue associated with tendency to false positives in recognition word list tasks. False recognitions are commonly defined as false memories or *confabulations,* a genuine memory dysfunction. However, other explanations could be taken into account. A recent paper clarified the distinction between *false recall*, as tendency to produce self-generated wrong responses, and *false alarms,* induced by external stimuli in specific contexts. Some evidences support the role of prefrontal regions, as ventrolateral prefrontal cortex, inferior frontal gyrus and anterior cingulate cortex, to avoid false alarms during cognitive tasks. Such regions offer specific contributions to executive processes involved in this behavior: inhibitory control and suppression of inappropriate information, strategic encoding, sustained attention, updating and control over interference driven by working memory. In this perspective, false recognitions can be interpreted as inaccurate commission of a response^30^.

Overall, our data have implication for routine clinical practice. A thorough neuropsychological assessment combining multiple cognitive measures is required to correctly define MCI, in order to identify those subjects requiring biomarkers assessment. In our cohort, the combined use of RAVLT and LM sub-measures discriminated patients with MCI due to AD from patients with MCI not related to AD. In the era of forthcoming disease-modifying therapies, which have the best chance to be effective the earlier they are applied, it is mandatory to identify AD patients in the prodromal phase of disease.

The present study has some limitations. It represents a retrospective investigation in a cohort of MCI patients subgrouped, according to the CSF profile, in MCI-AD (A+/T+ , n. 109) and non-AD MCI (A-/T-, n. 60). As expected, approx. 2/3 of patients referred to our Memory Clinic due to memory complaints, were affected by AD. We did not carry out specific statistical analysis according to single/amnestic or multi-domain MCI pattern. Only some quantitative measures from the two tests assessing verbal episodic memory were considered, in order to evaluate their predictive value on CSF AD profile; thus, qualitative parameters (e.g. *primacy* and *recency effect*, retroactive and proactive interference) were not included in the analysis.

In conclusion, in our study, the use of two specific tests of verbal episodic memory (RAVLT and LM) contributed to highlight the limits of the *one-test approach* for the correct evaluation of episodic memory deficits in MCI patients. Most importantly, the combined use of RAVLT and LM showed the best capacity to predict CSF AD profile. These results further support previous findings about the complementary and not interchangeable nature of the most widely used tests for episodic memory^18,40^.

In clinical practice, MCI patients showing altered delayed recall sub-measures at RAVLT and LM should undergo CSF analysis in order to rule out the presence of Alzheimer’s disease.

## Data Availability

The authors confirm that the data supporting the findings of this study are available within the article or its supplementary materials.

## References

[1] Petersen RC, Smith GE, Waring SC, Ivnik RJ, Tangalos EG, Kokmen E (1999). Mild cognitive impairment: Clinical characterization and outcome. Arch. Neurol..

[2] Petersen RC, Doody R, Kurz A, Mohs RC, Morris JC, Rabins PV (2001). Current concepts in mild cognitive impairment. Arch. Neurol..

[3] Petersen RC, Roberts RO, Knopman DS, Boeve BF, Geda YE, Ivnik RJ (2009). Mild cognitive impairment: Ten years later. Arch Neurol..

[4] Petersen RC, Aisen P, Boeve BF, Geda YE, Ivnik RJ, Knopman DS (2013). Mild cognitive impairment due to Alzheimer disease in the community. Ann. Neurol..

[5] Petersen RC (2004). Mild cognitive impairment as a diagnostic entity. J. Intern. Med..

[6] Winblad B, Palmer K, Kivipelto M, Jelic V, Fratiglioni L, Wahlund LO (2004). Mild cognitive impairment—beyond controversies, towards a consensus: Report of the International Working Group on Mild Cognitive Impairment. J. Intern Med..

[7] Tremont G, Miele A, Smith MM, Westervelt HJ (2010). Comparison of verbal memory impairment rates in mild cognitive impairment. J. Clin. Exp. Neuropsychol..

[8] Bondi MW, Edmonds EC, Jak AJ, Clark LR, Delano-Wood L, McDonald CR (2014). Neuropsychological criteria for mild cognitive impairment improves diagnostic precision, biomarker associations, and progression rates. J. Alzheimers Dis..

[9] Edmonds EC, Delano-Wood L, Clark LR, Jak AJ, Nation DA, McDonald CR (2015). Alzheimer's Disease Neuroimaging Initiative. Susceptibility of the conventional criteria for mild cognitive impairment to false-positive diagnostic errors. Alzheimers Dement..

[10] Edmonds EC, Delano-Wood L, Jak AJ, Galasko DR, Salmon DP, Bondi MW (2016). Alzheimer’s disease neuroimaging initiative. "Missed" Mild Cognitive Impairment: High False-Negative Error Rate Based on Conventional Diagnostic Criteria. J. Alzheimers Dis..

[11] Papaliagkas V, Kalinderi K, Vareltzis P, Moraitou D, Papamitsou T, Chatzidimitriou M (2023). CSF biomarkers in the early diagnosis of mild cognitive impairment and Alzheimer's disease. Int. J. Mol. Sci..

[12] Dubois B, Hampel H, Feldman HH, Scheltens P, Aisen P, Andrieu S (2016). Proceedings of the Meeting of the International Working Group (IWG) and the American Alzheimer's Association on “The Preclinical State of AD”; July 23, 2015; Washington DC, USA. Preclinical Alzheimer's disease: Definition, natural history, and diagnostic criteria. Alzheimers Dement..

[13] Jack CR, Bennett DA, Blennow K, Carrillo MC, Dunn B, Haeberlein SB (2018). NIA-AA research framework: Toward a biological definition of Alzheimer's disease. Alzheimers Dement..

[14] Sarazin M, Berr C, De Rotrou J, Fabrigoule C, Pasquier F, Legrain S (2007). Amnestic syndrome of the medial temporal type identifies prodromal AD: A longitudinal study. Neurology.

[15] Sarazin M, Chauviré V, Gerardin E, Colliot O, Kinkingnéhun S, de Souza LC (2010). The amnestic syndrome of hippocampal type in Alzheimer's disease: An MRI study. J. Alzheimers Dis..

[16] Dubois B, Feldman HH, Jacova C, Cummings JL, Dekosky ST, Barberger-Gateau P (2010). Revising the definition of Alzheimer's disease: A new lexicon. Lancet Neurol..

[17] Dubois B, Feldman HH, Jacova C, Hampel H, Molinuevo JL, Blennow K (2014). Advancing research diagnostic criteria for Alzheimer's disease: The IWG-2 criteria. Lancet Neurol..

[18] De Simone MS, Perri R, Fadda L, De Tollis M, Turchetta CS, Caltagirone C (2017). Different deficit patterns on word lists and short stories predict conversion to Alzheimer's disease in patients with amnestic mild cognitive impairment. J. Neurol..

[19] Dannhauser TM, Shergill SS, Stevens T, Lee L, Seal M, Walker RW (2008). An fMRI study of verbal episodic memory encoding in amnestic mild cognitive impairment. Cortex.

[20] Dickerson BC, Bakkour A, Salat DH, Feczko E, Pacheco J, Greve DN (2009). The cortical signature of Alzheimer's disease: regionally specific cortical thinning relates to symptom severity in very mild to mild AD dementia and is detectable in asymptomatic amyloid-positive individuals. Cereb. Cortex.

[21] Gour N, Ranjeva JP, Ceccaldi M, Confort-Gouny S, Barbeau E, Soulier E, Guye M, Didic M, Felician O (2011). Basal functional connectivity within the anterior temporal network is associated with performance on declarative memory tasks. Neuroimage.

[22] Wolk DA, Dickerson BC (2011). Alzheimer's Disease Neuroimaging Initiative: Fractionating verbal episodic memory in Alzheimer's disease. Neuroimage.

[23] Didic M, Barbeau EJ, Felician O, Tramoni E, Guedj E, Poncet M, Ceccaldi M (2011). Which memory system is impaired first in Alzheimer's disease?. J. Alzheimers Dis..

[24] Koric L, Ranjeva JP, Felician O, Guye M, de Anna F, Soulier E, Didic M, Ceccaldi M (2013). Cued recall measure predicts the progression of gray matter atrophy in patients with amnesic mild cognitive impairment. Dement. Geriatr. Cogn. Disord..

[25] Yonelinas AP, Ranganath C, Ekstrom AD, Wiltgen BJ (2019). A contextual binding theory of episodic memory: systems consolidation reconsidered. Nat. Rev. Neurosci..

[26] Braak H, Braak E (1991). Neuropathological staging of Alzheimer-related changes. Acta Neuropathol..

[27] Putcha D, Brickhouse M, Wolk DA, Dickerson BC (2019). Alzheimer's disease neuroimaging initiative. Fractionating the rey auditory verbal learning test: Distinct roles of large-scale cortical networks in prodromal Alzheimer's disease. Neuropsychologia.

[28] Price SE, Kinsella GJ, Ong B, Mullaly E, Phillips M, Pangnadasa-Fox L (2010). Learning and memory in amnestic mild cognitive impairment: contribution of working memory. J. Int. Neuropsychol. Soc..

[29] Balthazar ML, Yasuda CL, Cendes F, Damasceno BP (2010). Learning, retrieval, and recognition are compromised in aMCI and mild AD: are distinct episodic memory processes mediated by the same anatomical structures?. J Int. Neuropsychol. Soc..

[30] Festini SB, Katz B (2021). A frontal account of false alarms. J. Cogn. Neurosci..

[31] Rey, A. *L'examen clinique en psychologie.* [The clinical examination in psychology]. Presses Universitaries De France (1958).

[32] Carlesimo GA, Caltagirone C, Gainotti G (1996). The Mental Deterioration Battery: normative data, diagnostic reliability and qualitative analyses of cognitive impairment : The Group for the Standardization of the Mental Deterioration Battery. Eur. Neurol..

[33] Babcock, H., & Levy, L. *Test and Manual of Directions; The Revised Examination for the Measurement of Efficiency of Mental Functioning* (Stoelting, 1940).

[34] Novelli G, Papagno C, Capitani E, Laiacona M, Cappa SF, Vallar G (1986). Three clinical tests to research and rate the lexical performance of normal subjects. Archivio Di Psicologia Neurologia E Psichiatria..

[35] Brooks BL, Weaver LE, Scialfa CT (2006). Does impaired executive functioning differentially impact verbal memory measures in older adults with suspected dementia?. Clin. Neuropsychol..

[36] Wong CG, Jeffers SL, Bell SA, Caldwell JZK, Banks SJ, Miller JB (2022). Story memory impairment rates and association with hippocampal volumes in a memory clinic population. J. Int. Neuropsychol. Soc..

[37] Wicklund AH, Johnson N, Rademaker A, Weitner BB, Weintraub S (2006). Word list versus story memory in Alzheimer disease and frontotemporal dementia. Alzheimer Dis. Assoc. Disord..

[38] Perri R, Fadda L, Caltagirone C, Carlesimo GA (2013). Word list and story recall elicit different patterns of memory deficit in patients with Alzheimer's disease, frontotemporal dementia, subcortical ischemic vascular disease, and Lewy body dementia. J. Alzheimers Dis..

[39] Rabin LA, Paré N, Saykin AJ, Brown MJ, Wishart HA, Flashman LA (2009). Differential memory test sensitivity for diagnosing amnestic mild cognitive impairment and predicting conversion to Alzheimer's disease. Neuropsychol. Dev. Cogn. B Aging Neuropsychol. Cogn..

[40] De Simone MS, Perri R, Fadda L, Caltagirone C, Carlesimo GA (2019). Predicting progression to Alzheimer's disease in subjects with amnestic mild cognitive impairment using performance on recall and recognition tests. J. Neurol..

[41] Measso G, Cavarzeran F, Zappalà G, Lebowitz BD, Crook TH, Pirozzolo FJ (1993). The mini-mental state examination: Normative study of an Italian random sample. Dev. Neuropsychol..

[42] Giovagnoli AR, Del Pesce M, Mascheroni S, Simoncelli M, Laiacona M, Capitani E (1996). Trail making test: Normative values from 287 normal adult controls. Ital. J. Neurol. Sci..

[43] Appollonio I, Leone M, Isella V, Piamarta F, Consoli T, Villa ML (2005). The Frontal Assessment Battery (FAB): Normative values in an Italian population sample. Neurol. Sci..

[44] Monaco M, Costa A, Caltagirone C, Carlesimo GA (2013). Forward and backward span for verbal and visuo-spatial data: Standardization and normative data from an Italian adult population. Neurol. Sci..

[45] Ricci M, Pigliautile M, D'Ambrosio V, Ercolani S, Bianchini C, Ruggiero C (2016). The clock drawing test as a screening tool in mild cognitive impairment and very mild dementia: A new brief method of scoring and normative data in the elderly. Neurol. Sci..

[46] Basso A, Capitani E, Laiacona M (1987). Raven’s coloured progressive matrices: Normative values on 305 adult normal controls. Funct. Neurol..

[47] Morris JC, Ernesto C, Schafer K, Coats M, Leon S, Sano M (1997). Clinical dementia rating training and reliability in multicenter studies: The Alzheimer's Disease Cooperative Study experience. Neurology.

[48] Teunissen CE, Petzold A, Bennett JL, Berven FS, Brundin L, Comabella M (2009). A consensus protocol for the standardization of cerebrospinal fluid collection and biobanking. Neurology.

[49] Bellomo G, Indaco A, Chiasserini D (2021). Machine learning driven profiling of cerebrospinal fluid core biomarkers in Alzheimer’s disease and other neurological disorders. Front. Neurosci..

[50] Espinosa A, Alegret M, Valero S, Vinyes-Junqué G, Hernández I, Mauleón A (2013). A longitudinal follow-up of 550 mild cognitive impairment patients: Evidence for large conversion to dementia rates and detection of major risk factors involved. J. Alzheimers Dis..

[51] Hughes TF, Snitz BE, Ganguli M (2011). Should mild cognitive impairment be subtyped?. Curr. Opin. Psychiatry..

[52] Bradfield NI, Ames D (2020). Mild cognitive impairment: narrative review of taxonomies and systematic review of their prediction of incident Alzheimer's disease dementia. BJPsych Bull..

[53] Perri R, Serra L, Carlesimo GA, Caltagirone C (2007). Early Diagnosis Group of the Italian Interdisciplinary Network on Alzheimer's Disease. Amnestic mild cognitive impairment: Difference of memory profile in subjects who converted or did not convert to Alzheimer's disease. Neuropsychology.

[54] Wagner M, Wolf S, Reischies FM, Daerr M, Wolfsgruber S, Jessen F, Popp J, Maier W, Hüll M, Frölich L, Hampel H, Perneczky R, Peters O, Jahn H, Luckhaus C, Gertz HJ, Schröder J, Pantel J, Lewczuk P, Kornhuber J, Wiltfang J (2012). Biomarker validation of a cued recall memory deficit in prodromal Alzheimer disease. Neurology..

[55] Hildebrandt H, Haldenwanger A, Eling P (2009). False recognition correlates with amyloid-beta (1–42) but not with total tau in cerebrospinal fluid of patients with dementia and mild cognitive impairment. J. Alzheimers Dis..

[56] Haldenwanger A, Eling P, Kastrup A, Hildebrandt H (2010). Correlation between cognitive impairment and CSF biomarkers in amnesic MCI, non-amnesic MCI, and Alzheimer's disease. J. Alzheimers Dis..

[57] Rami L, Fortea J, Bosch B, Solé-Padullés C, Lladó A, Iranzo A (2011). Cerebrospinal fluid biomarkers and memory present distinct associations along the continuum from healthy subjects to AD patients. J. Alzheimers Dis..

[58] Rolstad S, Berg AI, Bjerke M, Blennow K, Johansson B, Zetterberg H (2011). Amyloid-β_42_ is associated with cognitive impairment in healthy elderly and subjective cognitive impairment. J. Alzheimers Dis..

[59] Bahar-Fuchs A, Villemagne V, Ong K, Chetélat G, Lamb F, Reininger CB (2013). Prediction of amyloid-β pathology in amnestic mild cognitive impairment with neuropsychological tests. J. Alzheimers Dis..

[60] Galluzzi S, Marizzoni M, Babiloni C, Albani D, Antelmi L, Bagnoli C (2016). PharmaCog Consortium. Clinical and biomarker profiling of prodromal Alzheimer's disease in workpackage 5 of the Innovative Medicines Initiative PharmaCog project: a 'European ADNI study'. J. Intern Med..

[61] Miotto EC, Brucki SMD, Cerqueira CT, Bazán PR, Silva GAA, Martin MDGM (2022). Episodic memory, hippocampal volume, and function for classification of mild cognitive impairment patients regarding amyloid pathology. J. Alzheimers Dis..

